# Minimum clinically important difference analysis confirms the efficacy of IgPro10 in CIDP: the PRIMA trial

**DOI:** 10.1111/jns.12204

**Published:** 2017-06-08

**Authors:** Ingemar S. J. Merkies, John‐Philip Lawo, Jonathan M. Edelman, Jan L. De Bleecker, Claudia Sommer, Wim Robberecht, Mika Saarela, Jerzy Kamienowski, Zbigniew Stelmasiak, Orell Mielke, Björn Tackenberg, Jean‐Marc Léger, on behalf of the PRIMA trial investigators, J. L. De Bleecker, W. Robberecht, M. Saarela, J. Franques, J.‐M. Léger, R. Juntas Morales, C. Sommer, A. Nguento, J. Schmidt, Ch. Schrey, J. Kamienowski, Z. Stelmasiak, G. Zwolińska

**Affiliations:** ^1^ Spaarne Hospital Hoofddorp, and Maastricht University Medical Centre Maastricht The Netherlands; ^2^ CSL Behring GmbH Marburg Germany; ^3^ CSL Behring LLC King of Prussia PA USA; ^4^ AZ St‐Lucas Gent Belgium; ^5^ Department of Neurology Universitätsklinikum Würzburg Würzburg Germany; ^6^ UZ Leuven Leuven Belgium; ^7^ Department of Neurology Helsinki University Central Hospital Helsinki Finland; ^8^ Dolnośląski Szpital Specjalistyczny Wroclaw Poland; ^9^ Samodzielny Publiczny Szpital Kliniczny Lublin Poland; ^10^ Department of Neurology Philipps University Marburg Germany; ^11^ Reference Center for Rare Neuromuscular Diseases Hôpital Pitié‐Salpêtrière, and University Paris VI Paris France; ^12^ AZ St‐Lucas Gent; ^13^ UZ Leuven Leuven; ^14^ HUS Meilahti Hospital Helsinki; ^15^ Hôpital de la Timone, Neurologie et Maladies Neuro‐Musculaire Marseille; ^16^ Groupe Hospitalier Pitié‐Salpêtrière Unité de Pathologie Neuro‐Musculaire Paris; ^17^ CHRU Hôpital Gui de Chauliac Montpellier; ^18^ Universitätsklinikum Würzburg Würzburg; ^19^ ASKLEPIOS Klinikum Uckermark GmbH Schwedt; ^20^ Universtitätsmedizin Göttingen, Georg‐August‐Universität Göttingen; ^21^ Facharzt für Neurologie Berlin; ^22^ Dolnośląski Szpital Specjalistyczny Wrocław; ^23^ Samodzielny Publiczny Szpital Kliniczny Lublin; ^24^ Centrum Neurologii Klinicznej Kraków

Dear Editor,

The PRIMA (PRivigen Impact on Mobility and Autonomy, NCT01184846) trial, a prospective, multi‐center, single‐arm, open‐label, phase III trial, was designed to assess efficacy and safety of IgPro10 (10% liquid IVIG formulated with L‐proline, Privigen®, CSL Behring, Berne, Switzerland) in patients with chronic inflammatory demyelinating polyneuropathy (CIDP) *(Léger et al.,* 2013 *)*. The primary outcome of the PRIMA study was the responder rate by the 10‐point adjusted Inflammatory Neuropathy Cause and Treatment (INCAT) disability score (responders defined as showing an INCAT score improvement ≥1 vs baseline). The success criterion (responder rate ≥35%) was met, making IgPro10 the second IgG product with demonstrated efficacy in CIDP (after IGIV‐C) *(Hughes et al.,* 2008 *; Léger et al.,* 2013 *)*.

Here we examine the clinical relevance of the PRIMA study results using the concept of minimal clinically important difference (MCID), which is defined as the smallest difference in clinical score that patients perceive as beneficial and that could lead to a change in the patient's management *(Jaeschke et al.,* 1989 *)*. For this analysis, responder rates for various outcome measures used in the PRIMA trial were recalculated based on MCID cut‐off values obtained through selected methods to determine whether the statistically significant results obtained previously also reflect clinically meaningful changes for patients with CIDP.

In the PRIMA trial, 28 adult patients with definite or probable CIDP were included. All the enrolled patients first received an IgPro10 induction dose of 2 g/kg body weight in week 1, followed by up to seven infusions of 1 g/kg body weight at 3‐week intervals.

Outcome measures used in the PRIMA trial were selected based on previous recommendations for assessment in inflammatory neuropathies *(Merkies and Lauria,* 2006 *; Lunn et al.,* 2008 *)*. Change in INCAT scores, Medical Research Council (MRC) sum scores, and maximum grip strengths upon treatment start recorded in the PRIMA trial (assessed at baseline and every 3 weeks thereafter *(Léger et al.,* 2013 *)*) were examined here by applying selected MCID‐based techniques *(Kleyweg et al.,* 1991 *; Hughes et al.,* 2001 *; Lunn et al.,* 2008 *)*.

Because of a lack of consensus on the optimal method for MCID determination in CIDP, a combination of techniques was recommended *(Merkies et al.,* 2010 *)*. The methods selected for the present analysis were an anchor‐based method (using the Short Form 36, question 2 *(Ware et al.,* 2000 *)*), which compares the changes in outcome measures with the patient's perception of clinical improvement, and a distribution‐based method that uses half standard deviation of each of the chosen scales *(Sloan et al.,* 2003 *)*.

The MCID cut‐off values, determined using the techniques described above and published previously, were adopted for INCAT score, MRC sum score, and grip strength *(Merkies et al.,* 2010 *)*. Because the INCAT and MRC sum scores only use integer values, the MCID for these parameters were rounded to 1 and 4, respectively. For grip strength assessment, an MCID value of 8 kPa was chosen because it has showed satisfactory discriminatory abilities between treatment and placebo outcomes in CIDP *(Merkies et al.,* 2010 *)*. In this analysis, all patients with a change in outcome measure between baseline and study end larger or equal to the MCID cut‐off value were considered responders.

From the results of this analysis, responder rates for all recalculated outcome measures showed that a substantial proportion of patients achieved a clinically relevant improvement. For the INCAT disability scale, the primary endpoint, the MCID‐based response rate was 61% (95% confidence interval [CI]: 42%, 76%; Fig. 1), higher than the preset level of >35%. Based on MRC sum score and grip strength MCIDs, 17 and 10 patients were defined as responders, which corresponds to response rates of 61% (95% CI: 42%, 76%) and 36% (95% CI: 21%, 54%), respectively.

**Figure 1:**
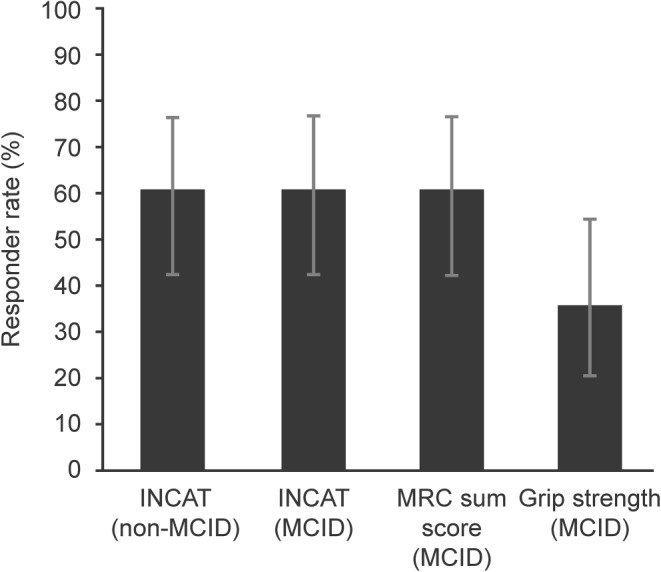
Error bars represent 95% confidence intervals. The responder rates for the PRIMA study (non‐MCID) were reported previously (Merkies et al., 2010 ).

In all patients except two, at least one of the outcome measures reached the calculated MCID cut‐off. Of the 28 patients 11 (39%) had at least two outcome measures that reached the calculated corresponding MCID cut‐offs, and in 8 patients (29%) all three scales reached the corresponding MCID thresholds.

The current analysis therefore demonstrates, by applying the concept of MCID, that the benefit of IgPro10 in CIDP is clinically meaningful in addition to being statistically significant. The proportion of patients reaching the predefined MCID cut‐off for the primary outcome (INCAT disability scale) was equivalent to the responder rate calculated in the original study *(Léger et al.,* 2013 *)*.

The findings using the INCAT scale were validated by the impairment outcome measures of MRC sum score and grip strength. The lower proportion of patients showing clinically meaningful improvement in grip strength (36%) compared with INCAT disability scale and MRC sum score (both 61%) is probably due to the following aspects. While grip strength evaluates focal impairment, in the current study of the dominant hand, the INCAT and MRC scores provide a more overall dysfunction of the patients examined *(Léger et al.,* 2013 *)*. In addition, the stringent cut‐off used for grip strength could have led to a lower MCID response when compared with the ordinal‐based INCAT and MRC measures, the scores of which might be inflated *(Tennant and Conaghan,* 2007 *; Marais and Andrich,* 2008 *; Léger et al.,* 2013 *; Vanhoutte et al.,* 2015 *)*. The small sample size and possibly non‐uniform improvement across muscle groups might have also contributed to the differences. Finally, local dependency is seen in MRC sum score, which could also inflate response findings *(Vanhoutte et al.,* 2012 *; Draak et al.,* 2015 *)*. The findings using the impairment measures were also compatible with previous reports *(Merkies et al.,* 2010 *)*.

The limitations of this analysis are related to methodological issues. First, the concept of MCID was applied to two outcome measures that are based on ordinal scales and are considered non‐linear (INCAT and MRC) *(Vanhoutte et al.,* 2013 *; Draak et al.,* 2014 *)*; therefore, the calculated MCID cut‐off values may vary across the range of values for these scales *(Merkies et al.,* 2010 *; Vanhoutte et al.,* 2013 *)*. Such variations in MCID have been demonstrated in several articles based on the varying measurement imprecisions (standard errors) *(Heesch et al.,* 2006 *; Hobart and Cano,* 2009 *; Vanhoutte et al.,* 2013 *)*. Second, the lack of consensus regarding which MCID determination technique (or combination thereof) should be used in CIDP warrants discussion among experts to reach a consensus. In this analysis, the anchor‐based method was considered appropriate to take into account both objective and subjective evaluation of improvement, while the distribution‐based method served as comparator. Third, the sample size is relatively small, as the power calculation was based on the results of the ICE trial and the expected response rate *(Léger et al.,* 2013 *)*. Briefly, due to the lower number of IVIG‐naïve patients in the PRIMA study compared with the ICE study, the responder rate was expected to be higher and the necessary sample size smaller (20 evaluable patients). In the ICE study, a similar number of patients in the IVIG‐C group was treated for 24 weeks (n = 33), while 23 patients not responding by week 6 were crossed over to placebo *(Hughes et al.,* 2008 *)*.

Despite these limitations, the findings in the current analysis demonstrate that the efficacy of IgPro10 in patients with CIDP shown in the PRIMA trial is clinically relevant.

## Disclosures

I. S. J. M. received funding for research from the Talecris Talents programme, the GSB CIDP Foundation International, and the European Union 7th Framework Programme (grant n°602273); furthermore, a research foundation at the University of Maastricht has received honoraria on his behalf for participation in steering committees of the Talecris ICE Study, CSL Behring, LFB, and Novartis. J.‐P. L. and J. M. E. are employees of CSL Behring. J. L. D. B. reports personal fees from CSL Behring for the PRIMA study conduct. C. S. reports support from CSL Behring for the PRIMA study conduct; personal fees from Astellas, Baxter, Genzyme, Pfizer, Grifols, Kedrion, and Pfizer; grants from the European Union 7th Framework Programme, outside the submitted work. W. R. reports grants from Von Behring Chair for Neuromuscular and Neurodegenerative Diseases, outside the submitted work. M. S. reports personal fees from Baxter, CSL Behring, Genzyme, Orion Pharma, and Pfizer, outside the submitted work. J. K. and Z. S. have nothing to disclose. O. M. is an employee of CSL Behring. B. T. reports personal fees from CSL Behring for study conduct; personal fees from Talecris/GRIFOLS and Octapharma, outside the submitted work. J.‐M. L. reports support from CSL Behring (PRIMA study), Novartis (CIDP study), LFB (MMN [Lime], and CIDP studies); personal fees from CSL Behring France and LFB, outside the submitted work.

## Belgium

J. L. De Bleecker, AZ St‐Lucas, Gent (five patients).

W. Robberecht, UZ Leuven, Leuven (three patients).

## Finland

M. Saarela, HUS Meilahti Hospital, Helsinki (three patients).

## France

J. Franques, Hôpital de la Timone, Neurologie et Maladies Neuro‐Musculaire, Marseille (two patients).

J.‐M. Léger, Groupe Hospitalier Pitié‐Salpêtrière Unité de Pathologie Neuro‐Musculaire, Paris (one patient).

R. Juntas Morales, CHRU Hôpital Gui de Chauliac, Montpellier (one patient).

## Germany

C. Sommer, Universitätsklinikum Würzburg, Würzburg (four patients).

A. Nguento, ASKLEPIOS Klinikum Uckermark GmbH, Schwedt (two patients).

J. Schmidt, Universtitätsmedizin Göttingen, Georg‐August‐Universität, Göttingen (one patient).

Ch. Schrey, Facharzt für Neurologie, Berlin (one patient).

## Poland

J. Kamienowski, Dolnośląski Szpital Specjalistyczny, Wrocław (three patients).

Z. Stelmasiak, Samodzielny Publiczny Szpital Kliniczny, Lublin (three patients).

G. Zwolińska, Centrum Neurologii Klinicznej, Kraków (two patients).
